# Treatise on the Poison of the Viper, on the American Poisons, on the Lauro-Cerasus, and on Some Other Vegetable Poisons. To Which Are Added, Observations on the Primitive Structure of the Animal Body; Different Experiments on the Reproduction of Nerves; and the Description of a New Canal of the Eye

**Published:** 1784

**Authors:** 


### LONDON MEDICAL JOURNAL,

For JAN. FEB. and MARCH, 1784.

### SECTION I.

### BOOKS.

I. *Traité ſur le Venin de la Vipere, ſur les Poiſons Americains, ſur le Laurier Ceriſe, et ſur quelques autres Poiſons vegetaux. On y a joint des obſervations ſur la ſtructure primitive du corps animal; differentes experiences ſur la reproduction des nerfs; et la deſcription d'un nouveau canal de l'œil.* i. e. *Treatiſe on the Poiſon of the Viper, on the American Poiſons, on the Lauro-Ceraſus, and on ſome other vegetable Poiſons. To which are added, Obſervations on the primitive ſtructure of the animal body; different experiments on the reproduction of nerves; and the deſcription of a new canal of the eye. By* Felix Fontana, *Natural Philoſopher to his R. H. the*

*Archduke grand duke of Tuſcany, and director of his cabinet of natural hiſtory.* 4to. Florence, 1781, 2 vols. with 10 copper-plates. Vol. I. 355 pages. Vol. II. 373 pages.

THESE two volumes are inſcribed to the academy of Upſal. The whole of the firſt and a conſiderable part of the ſecond volume are allotted to the poiſon of the viper. The obſervations on this ſubject are divided into four parts. Of theſe the firſt was publiſhed in Italian ſo long ago as the year 1765, and tranſlated into French ſoon after by M. Darcet. Something happened which delayed the publication of this tranſlation, and in 1776, the author coming to Paris, furniſhed M. Darcet with ſeveral additions and corrections, which were likewiſe tranſlated. The year following appeared a work on the uſe of the volatile alkali fluor as an antidote to the poiſon of the viper, in which are many aſſertions repugnant to what had been publiſhed ten years before by our author. The peruſal of this book induced him to make new experiments, and to this circumſtance we owe the ſecond, third, and fourth parts of his treatiſe. While the work was in the preſs, the ingenious and indefatigable author was was employed in varying and multiplying his experiments. Of the refults, which are related in a fupplement, we fhall give an account under their refpective heads.

The firft part of the treatife begins with a concife view of the opinions of former writers concerning the poifon of the viper. Before the time of Redi, we are told, it was not known in what this poifon confifts. It was he who firft difcovered the yellow fluid that renders the bite of the viper poifonous; but he was miftaken with regard to the feat of this fluid, which he fuppofed to be in the membrane that covers the canine teeth. This miftake was adopted more than half a century afterwards by James in his Dictionary, who has likewife copied all the erroneous opinions of Mead concerning the faline nature of the poifon.

This concife hiftorical view of the fubject is followed by an accurate defcription of the number, ftructure and ufe of the teeth of the viper. —The mechanifm by which the viper raifes its canine teeth when it bites, is allowed to be well defcribed by Nichols in the appendix to Mead's treatife on poifons. The virus iffues, it feems, from the point of each canine tooth, and not, as Redi fuppofed, from the membrane which

 envelops envelops theſe teeth and the other ſmaller ones at their baſis. Our author confirms the opinion ſuggeſted by Redi and adopted by Nichols, that the latter are intended to replace the canine teeth. —The virus iſſues from all the canine teeth at once. Nichols was wrong, therefore, in aſſerting, that it flows only from one tooth on each ſide at a time.—The receptacle of the virus is a little veſicle, which ſeldom contains more than three or four drops, and is ſituated under the muſcles at the ſide of the upper jaw. It is covered by a conſtrictor muſcle, which is ſo contrived that, however irritated the viper may be, the receptacle cannot be emptied by a ſingle bite. This veſicle terminates in a tranſparent excretory duct, which paſſes through the membrane that ſurrounds the baſis of each canine tooth, and diſcharges its contents thro' an almoſt imperceptible foramen at the fore part of the maxillary bone, which correſponds with another minute tube at the baſis of each canine tooth, ſo that the poiſon is conveyed into the body of the tooth, and is preſſed out through another hole at the point of the tooth.

Our author's experiments contradict Mead's aſſertion, that the bite of the viper is fatal to its own ſpecies. They likewiſe prove, that it is equally equally harmlefs to leeches and fnails, but eels, lizards, &c. die of it, and it likewife kills the tortoife, tho' not without difficulty.

This poifon is neither acid nor alkaline. It has no particular tafte, excites no inflammation on the tongue, nor does it leave any acrid or burning fenfation on the tongue like the poifon of the bee or the fcorpion. It is alfo without tafte or fmell when dried or powdered. Our author has met with no philofopher bold enough to fwallow any of this poifon; but his fervant, James Benvenuti, a Tirolefe, who was prevailed on to fubmit to this hazardous experiment, obferved nothing particular in its tafte only that it was flightly aftringent. It excites no pain when applied to the eyes, nor did it feem to occafion any pain when introduced into a wound in a dog. (Since this part of his work, however, was printed, the author, as he informs us in a fupplement, has found that the poifon of the viper kills animals very fpeedily when taken into the ftomach. This is contradictory to the opinion of the ancients, who thought that the poifon of ferpents was deftructive only when applied to a wound. *Non guftu fed in vulnere nocent*, fays Celfus; and Lucan, before him, makes Cato fay, *Morfu virus habent, & fatum dente*

*minantur*;

*minantur*; *pocula morte carent*). Two viper catchers at Pifa, who were bit, did not perceive the bite till the blood flowed from the puncture, and yet both thefe perfons were afterwards affected by the poifon. Thefe are proofs, our author thinks, that no acrid falts exift in the virus.

He thinks it probable that this fluid, which is fo poifonous to certain animals, may in the viper itfelf be effential to digeftion. It preferves its energy, we are told, for a long time, even for years, in a viper's tooth, without lofing its colour or tranfparency, and becomes as active as ever if the tooth is placed in warm water long enough to diffolve the poifon, which feems to be a gummy fubftance. Stopping up the orifice of the canine tooth with wax, which is faid to be a trick fometimes practifed by viper catchers, cannot always be done with impunity, as fome of the virus, inftead of paffing into the body of the tooth, may be preffed into the membranous fheath at its bafis.

The author next endeavours to afcertain the caufe of death in animals bit by the viper. It is not true, he obferves, that the globules of the blood (as they are improperly called) are decompofed by this virus. Convulfions, which animals animals of cold blood hardly ever experience, when bit by the viper, and thoſe of hot blood not always, are no proof that the virus contains cauſtic ſalts which irritate. Opium, tho' never ſuſpected of containing any ſuch ſalts, excites convulſions, and theſe ſymptoms, it is added, are not always the effects of irritation, but rather of the equilibrium between the antagoniſt muſcles being deſtroyed; and every one muſt have obſerved that animals, who die of hæmorrhage, die with horrid convulſions. He is of opinion that opium occaſions convulſions by deſtroying, in an irregular manner, the irritability of the muſcular fibre. He ſuppoſes the poiſon of the viper to be analogous in its effects to opium, and that, like mephitic air, it kills by deſtroying the irritability of the muſcular fibre and diſpoſing it to putrefaction. He thinks it probable that all the animal and vegetable poiſons act in a ſimilar manner. He obſerves that of all the venomous animals, hitherto known, none ſeems to poſſeſs ſo active a poiſon as the freſh-water polypus. In an inſtant it deprives worms of their living principle, of which they are otherwiſe ſo tenacious, being extremely irritable. Its mouth or its lips can hardly be ſaid to touch the worm, before the latter is dead, and and yet in this cafe no wound is inflicted, as the polypus has no teeth or other inftrument with which it can penetrate the fkin.

The ingenious Abbé here takes occafion to mention a fingular motion of the heart in a little microfcopic animal, which Lewenhoeck, fuppofing it to have wheels, has called *Rotifer*, or the *wheel polypus*. Thefe fuppofed wheels our author has found to be moveable arms, placed round the two trunks of the polypus, and which it moves with fo much quicknefs as to refemble a wheel. This fpecies of polypus is a gelatinous worm, which is commonly found in the earth or fand wafhed into the leaden gutters on the tops of houfes. But the moft curious circumftance in the hiftory of this little animal is the motion of its heart, which is very vifible with the microfcope, and appears to be abfolutely immoveable except when the animal moves its wheels or arms, and which it does only when it fwims. In this animal, therefore, the heart appears to be a voluntary mufcle, and during the greater part of its life is inactive; and yet this interruption of the circulation does not prevent its moving like other worms when its heart does not beat. He thinks that fome of his readers may doubt whether he has not miftaken the ftomach

ſtomach for the heart of the animal. This he allows to be poſſible, but he contends that, allowing it to be the ſtomach, the ſtructure of the animal will not be deemed leſs ſingular, as an organ like the ſtomach, ſubject to the will, is not known to exiſt in any other animal. He has dried a polypus of this ſpecies and kept it in this ſtate two years and a half, during which time it looked like a drop of dry glue, and yet when put into water it recovered its life and motion in two hours. He has likewiſe dried in the open air the *Seta equina*, or *Gordius* of Linnæus, till it had loſt almoſt the whole of its bulk and weight, and looked like a ſtraw that has been trampled on; and yet in leſs than half an hour, upon being put into water, this worm recovered its life and former ſize. He has ſince met with other little animals that have ſimilar properties. He means to ſpeak of all theſe in a work *on the life and apparent death of animals*. Very different, however, from all this it is, he adds, with the irritability which the muſcular fibres of animals loſe when poiſoned by the viper, for in theſe the principle of motion is loſt for ever.

In the ſecond part of his work the author treats of the effects of the poiſon of the viper on the different parts of animals. The experiments for theſe purpoſes were made on pigeons, guinea pigs, rabbits, cats, dogs, and frogs. A pigeon, bit by a viper in one of its legs, died in 12 minutes; a ſecond pigeon, bit by the ſame viper, died in 18; a third in 16; a fourth in 52 minutes, and a fifth in 20 hours; a ſixth was but little affected, and a ſeventh not at all, ſo that before the laſt was bit, the poiſon of the viper ſeems to have been totally exhauſted. He finds that an animal dies ſooner if bit in two places than if only in one; that the virus ſoon proves fatal to puppies, but that full-grown dogs generally recover tho' bit repeatedly by four vipers; that cats reſiſt the effects of this poiſon more than dogs, as in none of his trials did theſe animals, tho' bit by four and even ſix vipers, die, tho' they were much affected by the experiment: to produce death, he thinks, would require ten or twelve vipers.

Next follow experiments on the effects of the bite of the viper on different parts of animals. A tendon deprived of its ſheath, in a rabbit, did not ſeem to be affected by the poiſon. It is true, indeed, that the rabbit died ſoon after, but the expoſure of the tendon, we are told, is alone ſufficient to produce death in this animal.

In

In the courſe of this part of his work the author endeavours to aſcertain the quantity of virus requiſite to kill different animals. His experiments ſhew that $\frac{1}{1000}$ of a grain, introduced into a muſcle through a wound, proves fatal to a ſparrow ; and that five or ſix times this quantity will infallibly kill a pigeon. The ſparrows, on which he made his experiments, weighed ſomewhat leſs than an ounce each, and the pigeons rather more than ſix ounces. On this ground, therefore, he ſuppoſes that to produce a ſimilar effect in an ox, allowing its weight to be 750 pounds, 12 grains of the virus will be required, and 3 grains in a man, whoſe weight is about 150 pounds. He is of opinion that the effect of the virus is proportioned to its quantity. He obſerves that a viper, of a moderate ſize, contains only two grains of virus in its veſicle, but as the animal gives out only a ſmall part of its poiſon at each bite, he conjectures that twenty vipers would be required to kill an ox, and five or ſix to produce the ſame effect in a man.

In the third part of the work our author attempts to aſcertain the length of time the virus requires to produce its effects. For this purpoſe he cut off the legs of ſeveral pigeons and frogs, and theſe parts were bit the moment they were amputated, but without any effect. In others the limb was cut off immediately after the bite, but the reſult was the ſame in theſe as in the preceding experiments; a proof that the virus requires a certain time to produce its effects, and this period, we are told, is found to vary according to the ſize of the animal.

We are next preſented with ſome inquiries concerning the action of the poiſon on the blood of animals. The poiſon of two vipers injected into the jugular vein of rabbits inſtantly produced convulſions, and in leſs than two minutes, death. On diſſection the blood in the heart and great veſſels was found coagulated. In one inſtance, indeed, it failed to produce theſe effects, and in another the animal lived ſeveral hours; but in both theſe the author acknowledges he was not ſufficiently accurate with his injection.

It appears from a great number of experiments that the virus produces no effect when applied to the nerves; and he is led to imagine, that the common ſymptoms of what are called nervous diſorders are doubtful and fallacious; that they may exiſt without any apparent diſorder of the nerve, and that a ſimple alteration in the ſtate of the blood is ſufficient to excite all theſe phænomena phænomena and that in an inſtant. He concludes that if Mead had been aware that a very ſmall quantity of the poiſon of the viper kills inſtantaneouſly when injected into the blood, and yet is perfectly innocent when applied to the nerves, he would certainly not have had recourſe to animal ſpirits and to the nerves, to explain the action of the poiſon on animals.

When our author aſſerted in the firſt part of his work, that the poiſon kills by deſtroying irritability, he was not aware of theſe circumſtances; viz. its effects on the blood, and its inefficacy when applied to the nerves. He now thinks this hypotheſis muſt in part be modified, as this loſs of irritability is rather an effect than a cauſe, and is a conſequence of the change produced in the blood by the virus rather than an effect of the virus on the muſcular fibre.

In the fourth part of his treatiſe he examines the effects of the different remedies that have been hitherto recommended againſt the bite of the viper.

His inquiries, relative to the quantity of virus requiſite to produce death in different animals, render it, as we have already ſeen, extremely doubtful whether it ever proves fatal to the human ſpecies. Of the perſons who are bit by the the viper it is very rare, our author obſerves, to hear of two who have uſed the ſame remedy, and yet they all recover; and from this circumſtance he argues, that a diſeaſe which gives way to every remedy, even to oppoſite ones, can never be dangerous. He himſelf has ſeen ten or twelve inſtances, and has heard of fifty others, of perſons bit by the viper, only one of whom died, and in this caſe deep ſcarifications, which produced gangrene, were made in the patient's arm. He quotes a few caſes from authors, which render it probable that even the bite of a rattle-ſnake is not always fatal to man.

Concerning the remedies hitherto employed he obſerves, that De Juſſieu, who, on Mead's authority, ſuppoſed the nature of the poiſon to be acid, employed the volatile alkali, or *Eau de Luce*, as an antidote to it. Our author finds this to be a remedy of no efficacy in theſe caſes, and the reputation which this and ſome other means of cure have acquired, he aſcribes to the circumſtance of the bite of the viper being not always fatal to the human ſpecies. In ſome of his experiments he immerſed the wounded part in hot oil of turpentine, and in hot water. Both theſe applications ſeemed to leſſen the inflammation. Having obſerved that dogs and cats recovered recovered fooneft when they vomited moft, he was induced to give emetic tartar to dogs that had been bit. Of the vomiting excited by this method he obferves, that altho' it is certainly not a means of cure on which we can depend, yet he will not fay that it is altogether ufelefs. Cantharides applied to the wound conftantly did harm by accelerating the local inflammation, and when given internally was, like the emetic, of doubtful efficacy. The Peruvian bark was tried but without any real advantage.—Scarification produced the fame bad effect as Cantharides.—Theriaca applied to the wound was of no ufe; and common oil, tho' formerly extolled by Mead as a remedy employed by viper-catchers, proved equally inefficacious.—Leeches, and fuction by the mouth, did not in the leaft retard the operation of the poifon.—He next tried the effect of amputation, and he found that guinea pigs, who were bit in the leg, generally recovered when the amputation was performed in lefs than fix minutes after the bite. A rabbit, bit in its ear, was no more affected than another, whofe ear had been cut off without being bit. In other rabbits the fame thing was obferved at the end of fix, and in a puppy at the end of twenty minutes. He now attempted to determine determine the effect of biting the ſkin and then cutting out the part; and he found in ſeveral experiments with rabbits and dogs, that the animals eſcaped the uſual effects of the poiſon if the exciſion was performed within twenty minutes after the bite.

Having proved that the poiſon is communicated, not by the nerves, but by the blood, he began to think that a method of preventing its effects, eaſier and leſs painful than amputation, might be adopted; viz. a ligature applied tight enough to ſtop the circulation in the limb. As pigeons are eaſily affected by the poiſon he preferred theſe for the experiment. Immediately after one of theſe had been bit in the leg, a ribband was tied cloſe round the limb. The local affection ſoon began to appear. The limb ſwelled, became livid and approaching to ſphacelus. At the end of about ten hours the ligature was removed, and the limb gradually recovered its natural appearance. The reſult was the ſame in five other ſimilar experiments, and this our author conſiders as a proof that the virus, after a certain time, loſes its efficacy. He has ſince found, that this method, tho' it ſucceeds with pigeons and guinea pigs, is generally ineffectual in rabbits. He does not think it right, right, however, to reject it as absolutely useless. Of its effects in man he has had no experience. He observes that it will be unnecessary to make the ligature very tight, or to continue it long on the limb, as he has repeatedly seen the experiment succeed equally well in pigeons, when the bandage was so loose as not to intercept the circulation, and was removed at the end of an hour.

In an appendix to these inquiries the author gives us the result of his experiments on three other remedies; viz. the *pierres de Cobras* (which have been extolled by Abbé Teemeyer, in the *Raccolta di Opuscoli Scelti* of Milan as an antidote to the poison of the viper), Theriaca, and Quicklime. The first of these was found to be no other than calcined hartshorn, and of no efficacy. Indeed it is observed of the Abbé Teemeyer, that he is a credulous writer, as he has recommended the same remedy in cases of hydrophobia, and advises a sea horse's tooth to be worn in the pocket as a specific against the poison of the viper. Kæmpfer's method of cure, with theriaca and scarifications, has also been tried by our author without any good effect. Quicklime applied to the wound, in pigeons, seemed to be of use. A similar effect was produced by absorbent earths. He lays no great stress, however, on either of these remedies, as the animals did equally well when nothing was applied to the wound.

In the course of his experiments on this subject, he has repeatedly seen good effects produced by lunar caustic applied to the wound, and given internally, at the same time, in solution. Rabbits, we are told, were in general cured by this method; but a greater variety of experiments must be made with it in different animals before we can determine what degree of confidence can be placed in it as a remedy.

The treatise on the poison of the viper is followed by two essays, the first of which relates to the *Ticunas*, an American poison, and the second to laurel-water. Both these papers have already been printed in the *Philos. Trans.* Vol. LXVIII. He now adds a second essay on the latter of these poisons. When his former observations were communicated to the Royal Society, he was unable to determine whether the essential oil of the Lauro-cerasus was of a poisonous nature; but his experiments have since proved it to be no less fatal than the spirit, and also that the latter preserves its destructive qualities after being almost wholly deprived of its odorous and and ſapid parts. The eſſential oil, when dried, becomes a reſinous ſubſtance, and even in this ſtate preſerves its energy.

As a proof of the extreme activity of this oil, experiments are related, which ſhew that it kills when applied only to the inſide of the mouth, and of courſe without being ſwallowed. At firſt, from ſome trials which he has ſince found were not ſufficiently accurate, he was led to think, that it might be injected into the veins of animals with impunity; but he has ſince been convinced that it proves inſtantly fatal in this way.

It is poiſonous to vipers, ſerpents, and pigeons, not only when ſwallowed, but alſo when applied to a wound. It proves deſtructive to pigeons, when applied only to the eye, and when applied to the heart inſtantly deprives it of its irritability. Notwithſtanding theſe properties which render the oil of the Lauro-ceraſus one of the moſt terrible and fatal poiſons hitherto diſcovered, we are told that in Italy it has been publicly ſold by perfumers under the name of eſſence of bitter almonds, and that many perſons are in the habit of mixing it with their ragouts. The preſent Grand Duke of Tuſcany, it ſeems, has wiſely ſuppreſſed this dangerous abuſe in his dominions.

After theſe obſervations and experiments on the poiſon of the Lauro-ceraſus, the author preſents us with the reſults of his trials with the Toxicodendron, which has generally been deemed a fatal poiſon, tho' ſome writers have denied that it poſſeſſes any ſuch property. In examining this vegetable he himſelf was thrice poiſoned by its leaves. The juice and extract of the plant were given to different animals without any effect; but a woman, who in gathering the leaves had ſeen two little drops of the juice fall on the back of her hand, and who, three days after, obſerved three minute ſpots on this part, was ſeized on the ſixth day with a ſwelling of her face, neck, throat, and hands, the ſkin of which became ſo inflamed and painful as to confine her to her bed fifteen days, at the end of which time the Epidermis peeled off. Our author was induced to conſider this as an accidental diſeaſe, eſpecially as he had applied the juice to the bare ſkin of rabbits and pigeons, had given it them with their food, and had even introduced it into wounds in thoſe animals without the leaſt effect. He was ſtill more confirmed in this opinion, when he had poured ſeveral large drops of the juice on the hands of two gardeners, who indeed obſerved the black ſpots three days after as in the other caſe, but experienced no other effect from the experiment. Our author now ventured to try it on himſelf. He accordingly touched the back of his hand with a ſmall drop of the juice, and it produced nearly the ſame effect as in the woman. The Epidermis was in many places diſtended with a watery fluid, and thirty days elapſed before the effects of the poiſon were entirely removed. At the end of that period, when he thought himſelf cured, he was induced to make ſome experiments on the air of the leaves, and in ſo doing he could not avoid touching them, but as they were entire he thought there could be no danger in this. Six days after this he began to ſwell again in the ſame manner as before; but this ſecond attack was neither ſo violent nor of ſo long duration as the firſt. At the expiration of three weeks he again ventured to touch the leaves, and in four days he experienced another ſlight repetition of the ſame ſymptoms as before. The gardeners, he thinks, owed their ſafety to their callous hands.

Next follows an account of ſome experiments with the oil of tobacco. In pigeons, this oil uſually excited vomiting when applied to a wound, and the limb to which it was applied was was ſometimes deprived of motion. This laſt effect, our author thinks, might be accidental. In no inſtance did it produce death.

We come now to ſome obſervations on the ſtate of the nerves in diſeaſes. Hoffman in his *Medicina Rationalis*, our author remarks, and, more lately, Muſgrave, have been inclined to conſider all diſeaſes as nervous. Some phyſicians, it is added, have ſuppoſed them to be hardened and dry, others flaccid and relaxed in a morbid ſtate; but his experiments having led him to obſerve, that there are poiſons which produce no effect when applied to a nerve, and yet excite all the ſymptoms uſually called nervous, when injected into the blood or applied to a muſcle; he doubts whether our opinions on this ſubject are well grounded, eſpecially as he has remarked that in frogs the motion of the heart is not affected by irritating the nerves. He doubts alſo whether nervous medicines, as they are called, act upon the nerves. He is aware that opium, when ſwallowed, has been thought to operate in this way; but who will venture to aſſert, ſays he, that it is not acted upon by the gaſtric juice and abſorbed? It has been argued indeed by Dr. Monro, of Edinburgh (*Eſſays Phyſ. and Lit.* Vol. 3.) that a ſolution of opium in ſpirits of of wine, applied to the nerves of frogs, produced palſy; but our author, after repeating and varying the experiment, aſſerts that this effect is due ſolely to the ſpirit of wine, as no ſuch conſequence enſued when a watery ſolution of opium was made uſe of. His experiments have convinced him that the blood is the vehicle of this as well as of the other poiſons.

The author next preſents us with experiments on the reproduction of the nerves. That a nerve, when divided, is capable of being reunited is a curious fact, for which anatomiſts are indebted to Mr. Cruikſhank, who after having divided a nerve of the eighth pair in a dog, took out about an inch of the nerve, and found at the end of a certain time that the ſpace was filled up with an irregular ſubſtance. The late Dr. Hunter doubted whether this was a real regeneration of the nerve, as did our author when he ſaw it in 1778, particularly as he had often divided the ſciatic nerve in animals without obſerving any ſuch appearance. To ſatisfy his doubts on this ſubject, he divided the ſciatic nerves of twelve rabbits, and in ſix of them he cut out ſix or eight tenths of an inch of the nerve. Some of theſe animals lived thirty days after the operation, but in none of them did there appear any mark mark of nervous reproduction. His experiments, however, did not ftop here. He now either divided or took out a portion of the *par vagum*, and of the intercoftal nerve, in twenty-four other rabbits, and in thirteen of thefe he afterwards obferved fuch a reunion as had been fhewn him by Mr. Cruikfhank. He examined the uniting fubftance with his microfcope, and he was convinced that it was real nerve, as he could trace the continuation of the fpiral lines and white bands he has obferved in other nerves. He does not venture to affert, however, that the reproduction is perfect, that is, that the cylinders, of which he fuppofes the nerves to be compofed, are continued through it without interruption. He obferves that a divifion of the phrenic nerve is perhaps the only experiment that can decide this matter. If, in this cafe, the reunion were to take place, the nerve, if irritated above the junction, would excite the action of the diaphragm. This experiment, for want of fufficient leifure, our author has not yet made, nor has he as yet afcertained what other nerves, befides the eighth pair and the intercoftal, poffefs this power of reproduction. The failure of the experiment when the fciatic nerves were divided, he thinks, might be owing to the great motion of the of the part. After all, however, we are inclined to believe that the reunion in queſtion is brought about merely by the intervention of cellular ſubſtance.

Theſe experiments are followed by obſervations on the primitive ſtructure of the animal body. Our ingenious and reſpectable author, during his reſidence in London, having read ſome account of Dr. Monro's ſuppoſed diſcoveries in the Edinburgh Medical Commentaries, wrote two very polite letters to the learned profeſſor, to requeſt ſome farther information on the ſubject. One of theſe letters was ſent by the poſt, the ſecond was delivered by the hands of a friend; but no anſwer was returned to either.

Our author began his inquiries with examining the nerves as they appear to the naked eye, when diveſted of their ſheath. Viewed in this manner they exhibited an appearance of ſpiral bands, like a ribband twiſted round a cylinder. Theſe bands appeared very diſtinct, and uniformly the ſame in every nerve, when viewed through a lens that magnified only ſix times; but when obſerved with a microſcope of much greater magnifying powers than this, the ſpiral bands diſappeared, and in their ſtead were ſeen parallel and twiſted fibres extending through

 the the whole courſe of the nerve. At length, after repeated obſervations, he found that with a ſtronger or a weaker light he could, with the ſame lens, have the appearance either of ſpiral bands or twiſted fibres. He now conſidered the former of theſe as an optical deception, and was perſuaded that the nerves are compoſed of twiſted fibres.

He was now deſirous to determine whether theſe fibres are ſolid or hollow. For this purpoſe he divided a nerve, and viewing it through a lens that magnified 500 times, he could diſtinguiſh ſeveral minute tranſparent cylinders, and having confirmed, as he thinks, this fact by repeated and numerous obſervations, he ventures to aſſert that a nerve is compoſed of a great number of tranſparent, homogeneous, uniform, very ſimple cylinders, which ſeem to be formed of a very ſubtile tunic, filled with a tranſparent gelatinous fluid that is inſoluble in water. The cylinders, he is convinced, are the primitive organic elements of the nerves.

He next treats of the ſtructure of the brain, and deſcribes the appearance of the cortical and medullary ſubſtances, and retina, when viewed through a lens of great magnifying powers. The appearances exhibited by the tendons, tendons, when viewed in the ſame manner, are alſo deſcribed. In examining the diaphragm of a rabbit through a microſcope, he found that not the leaſt filament of a nerve paſſes into its tendinous part, which is a proof, he thinks, that the tendons are deſtitute of nerves, and of courſe are compoſed of a ſubſtance different from the muſcles. The tendinous and fleſhy fibres, he obſerves, are intimately connected; but the fleſhy fibre, he contends, never becomes tendinous or the tendinous fibre fleſhy. He deſcribes the difference between the nervous, muſcular, and tendinous ſubſtances when viewed through a microſcope, and ſpeaks highly of Prochaſka's work *De Carne Muſculari*, printed at Vienna in 1778; a performance, he ſays, which has left us but little to wiſh for on this ſubject.

Our author offers ſome reflections on muſcular motion; but at the ſame time acknowledges the uncertainty of our knowledge on this head. He is inclined to conſider it as an electrical phænomenon, at leaſt, he thinks our diſcoveries concerning the Gymnotus and Torpedo render this, if not probable, at leaſt poſſible.

He next deſcribes the twiſted fibres of the cellular ſubſtance and membranes, and likewiſe of the hair, epidermis, nails, bones, and fat of the animal body. He likewiſe deſcribes theſe fibres as they appeared in ivory, foſſils, &c. when examined with a microſcope. All theſe different appearances are delineated in ſeveral very elegant engravings.

The author in this part of his work takes occaſion to enter pretty fully into the ſubject of microſcopic errors, and mentions the means he has uſed to avoid them; and yet notwithſtanding his care and ingenuity it ſeems certain, that the objects he has delineated are like the appearances of the ſame kind which we have lately had occaſion to mention (Vol. IV. p. 130) founded wholly on optical illuſion.

Theſe microſcopical inquiries are followed by a letter written in 1778 to Dr. Adolphus Murray, profeſſor of anatomy at Upſal, and containing a ſhort deſcription of a new canal which our author has diſcovered in the eye. This canal is formed by the *ligamentum ciliare*, or rather is ſurrounded by that ligament. Its internal ſurface is ſmooth and equal, and it may be eaſily detached from the *ſclerotica*. He has ſeen both water and mercury paſs from one ſide to the other of it, but as yet he is not able to ſay any any thing of its ufe, or of the tranfparent fluid with which it is moiftened.

We have now gone through the whole of this valuable and laborious performance, which contains the refult of upwards of *fix thoufand* experiments, and we have beftowed on it that degree of attention which we think its importance entitles it to. The author modeftly concludes it with acknowledging his readinefs to liften to criticifm and correction, but at the fame time protefts againft thofe pretended philofophers, who oppofe poffibilities and fcholaftic prejudices to facts; and he thinks it right to add, that as his experiments have been numerous, a fmall number of different refults will, in his opinion, not be fufficient to invalidate his affertions.

